# A Proposed Framework for Early Prediction of Schistosomiasis

**DOI:** 10.3390/diagnostics12123138

**Published:** 2022-12-12

**Authors:** Zain Ali, Muhammad Faisal Hayat, Kamran Shaukat, Talha Mahboob Alam, Ibrahim A. Hameed, Suhuai Luo, Shakila Basheer, Manel Ayadi, Amel Ksibi

**Affiliations:** 1 Department of Computer Engineering, University of Engineering and Technology, Lahore 54890, Pakistan; zain.zyan@gmail.com (Z.A.); muhammad.faisal.hayat@uet.edu.pk (M.F.H.); 2 School of Information and Physical Sciences, The University of Newcastle, Newcastle, NSW 2308, Australia; suhuai.luo@newcastle.edu.au; 3 Department of Data Science, University of the Punjab, Lahore 54890, Pakistan; 4 Department of Computer Science and Information Technology, Virtual University of Pakistan, Lahore 54000, Pakistan; talhamahboob95@gmail.com; 5 Department of ICT and Natural Sciences, Norwegian University of Science and Technology, 7034 Trondheim, Norway; 6 Department of Information Systems, College of Computer and Information Science, Princess Nourah bint Abdulrahman University, P.O. Box 84428, Riyadh 11671, Saudi Arabia; sbbasheer@pnu.edu.sa (S.B.); mfayadi@pnu.edu.sa (M.A.); amelksibi@pnu.edu.sa (A.K.)

**Keywords:** machine learning, Schistosomiasis, healthcare data, data imbalance, feature selection, data resampling, SMOTE, artificial intelligence

## Abstract

Schistosomiasis is a neglected tropical disease that continues to be a leading cause of illness and mortality around the globe. The causing parasites are affixed to the skin through defiled water and enter the human body. Failure to diagnose Schistosomiasis can result in various medical complications, such as ascites, portal hypertension, esophageal varices, splenomegaly, and growth retardation. Early prediction and identification of risk factors may aid in treating disease before it becomes incurable. We aimed to create a framework by incorporating the most significant features to predict Schistosomiasis using machine learning techniques. A dataset of advanced Schistosomiasis has been employed containing recovery and death cases. A total data of 4316 individuals containing recovery and death cases were included in this research. The dataset contains demographics, socioeconomic, and clinical factors with lab reports. Data preprocessing techniques (missing values imputation, outlier removal, data normalisation, and data transformation) have also been employed for better results. Feature selection techniques, including correlation-based feature selection, Information gain, gain ratio, ReliefF, and OneR, have been utilised to minimise a large number of features. Data resampling algorithms, including Random undersampling, Random oversampling, Cluster Centroid, Near miss, and SMOTE, are applied to address the data imbalance problem. We applied four machine learning algorithms to construct the model: Gradient Boosting, Light Gradient Boosting, Extreme Gradient Boosting and CatBoost. The performance of the proposed framework has been evaluated based on Accuracy, Precision, Recall and F1-Score. The results of our proposed framework stated that the CatBoost model showed the best performance with the highest accuracy of (87.1%) compared with Gradient Boosting (86%), Light Gradient Boosting (86.7%) and Extreme Gradient Boosting (86.9%). Our proposed framework will assist doctors and healthcare professionals in the early diagnosis of Schistosomiasis.

## 1. Introduction

Schistosomiasis, snail fever or bilharzia, is one of the most lethal and contagious among several ignored tropical diseases of the world. This parasitic disease affixed to the skin through defiled water enters the human body. It moves into human veins, where parasites lay eggs which becomes the reason for two stages (chronic and acute) of Schistosomiasis [1,2]. The most severe type of late-stage Schistosomiasis japonica in Asia is advanced Schistosomiasis. Hepatosplenomegaly, portal hypertension, gastroesophageal varices, periportal and liver cirrhosis, and other significant liver parenchyma damage are all caused by parasites trapped in eggs [3,4]. Advanced Schistosomiasis patients have a high rate of fatality or disability. World health organisation (WHO) reported that millions of people got infected [5]. There are over 30,000 advanced Schistosomiasis patients in China, and the number of cases has grown in recent years [6].

The quantity and size of medical data are increasing continuously. Because of this enormous volume of information, the prognosis of diseases, analysis based on statistics, extraction of disguised information, and identification of symptoms causing the disease are now possible in the digital era [7,8]. The significant data mining and machine learning techniques can help in the early prediction of Schistosomiasis, which can be very helpful for decision-makers and researchers [9]. However, dealing with a huge volume of data such as images, historical data, and genes-related expressions has been the main problem in data mining and machine learning applications which requires high computational time and storage capability that leads to poor performances. Feature selection plays an important role in solving the issue of huge data. It can be applied to reduce the dimension, irrelevance, and redundant data features, shorten the training time and improve performance [10].

A comprehensive model is not only beneficial to health professionals, but it may also assist people in diagnosing which factors may harm their health. The fundamental objective for early prediction of Schistosomiasis is to use medical data to assist medical practitioners in disease diagnosis, reduce mortality, and improve recovery. Statistical approaches are used in several risk prediction models, including K-nearest neighbour, discriminant analysis, and logistic regression [11]. Classification and regression models were also used to forecast risk variables with the progress of machine learning and artificial intelligence technology [12]. Machine learning and pattern recognition algorithms have demonstrated promising performance for high-dimensional and imbalanced clinical datasets. A dataset can be imbalanced when one class is over-represented among others. This concern is especially fundamental in real-world datasets where it’s expensive to misclassify models from the minority class, such as detection of fake calls, diagnosis of diseases like Schistosomiasis, data recovery, and text categorisation. Machine learning algorithms are a collection of approaches that automatically discover patterns from huge, complex data as an alternative to traditional prediction methods [13,14,15,16].

Data mining approaches were integrated with various critical parameters, such as clinical, socioeconomic and geographical factors, to diagnose Schistosomiasis. Various computational approaches are commonly used to uncover the link between various illnesses and patient clinical characteristics. Furthermore, machine learning techniques to find significant features from complicated datasets demonstrate their value. On the other hand, schistosomiasis pathogenesis is a complex process influenced by various factors such as demography, socioeconomic situation, and healthcare infrastructure. Therefore, an appropriate analytical framework has been required to combine this multidimensional, unstructured, and collinear data. This study aims to create a framework based on machine learning models for early Schistosomiasis prediction. We employed various computational methods, machine learning models and feature selection techniques to predict Schistosomiasis. The main contributions of our work are:

- A latest and imbalanced dataset related to Schistosomiasis has been employed for this research.
- Preprocessing like missing values imputation, data transformation, feature selection techniques correlation. Information gain, gain ratio, ReliefF, and OneR minimise the features for prediction and resampling techniques, Random undersampling and oversampling, Cluster Centroid, Near miss, and SMOTE have been employed to carter the problems in the dataset.
- Advanced machine learning techniques have been employed, which outperformed state-of-the-art methods.
- Our proposed framework can aid medical and healthcare practitioners in the early identification and improved treatment of Schistosomiasis.

The remainder of this work is structured as follows. Section 2 reviews the literature, including past work on Schistosomiasis and traditional statistical approaches applied to high-dimensional medical data. Section 3 describes the methodology that contains dataset description, data preprocessing, feature selection, resampling, data transformation approaches, and modelling. Section 4 details the framework’s experimental setup and outcomes. Section 5 discusses the ramifications of the results in terms of their relevance. Section 6 finishes the report by highlighting future work in this field.

## 2. Literature Survey

Li et al. divided Schistosomiasis cases into two groups: favourable and poor prognoses [1]. The cases in which improved patient health are referred to as favourable prognoses, while cases of perishing and death are classified as poor prognoses. Various machine learning models were employed for advanced Schistosomiasis prediction in which ANN outperformed the other models. Holing et al. [17] proposed a method for predicting 1-year unfavourable prognosis for advanced Schistosomiasis. Demographics, clinical factors, medical exams, and test results were used to choose candidate predictors. To build one-year prognostic model, five machine learning techniques were used: Logistic Regression (LR), Decision Tree (DT), Random Forest (RF), Artificial Neural Network (ANN), and Extreme Gradient Boosting (XGBoost). The model’s performance was assessed using the area under the receiver operating characteristic curve (AUROC). XGBoost has outperformed other Machine learning models based on performance.

Computational techniques to estimate the amount of Schistosomiasis susceptibility and vulnerability, the researchers Olanloye et al. [18] compared support vector machine (SVM) models, i.e., Linear, Quadratic, Cubic, Fine Gaussian, and Medium Gaussian and Coarse Gaussian. Receiver Operating Character (ROC) and Parallel Coordinate Plots (PCP) were used to evaluate the experiments in terms of accuracy, processing speed, and execution time. Finally, it was determined that Medium Gaussian is the best among the six models. Asarnow et al. [19] applied the Asarnow-Singh algorithm to a set of images and extracted features by defining a threshold to identify Schistosomiasis mansoni on images with infected foreground and parasitic background areas. Effective results were obtained by using SVM for training and testing images. Data was gathered through a network of wireless sensors, and Kasse et al. [20] developed a system based on IoT monitoring that can help control and predict disease. For disease detection and transmission, multiple data mining algorithms were applied. The SVM detects irregularity better than other models. Chicco et al. [21] collected a dataset of 324 patients, of which 96 were mesothelioma patients. The imbalanced class problem arises because of the ratio between the positive and negative instances. The perceptron-based neural network was used to check the effectiveness of ANN. RF feature selection (RFFS) was used to investigate the most relevant features due to the best diagnosis predictive results.

According to the previous literature, the problem of class imbalance with medical datasets exists, and there are a couple of articles regarding this matter. Subsequently, significant writing looked at clinical science and different fields of science. Different methods to solve class imbalance were described [22]. Various techniques to solve this problem are categorised into different levels: data, algorithmic, cost-sensitive, feature selection and ensemble level. Understanding which preprocessing strategy should be chosen according to the problem is challenging. The dataset is considered to be an imbalanced dataset in which instances between both classes are not equal. Therefore, the class imbalance is mostly removed at the data level, and multiple under and oversampling-based approaches can be used.

Under-sampling was not preferred to deal with class imbalance by some scholars [23] [24], while others do not prefer oversampling [25]. On the other hand, other studies [26] show that undersampling, especially multiple undersampling, performs better. In the dataset, data preprocessing techniques could remove missing values, redundancy, and high scarcity. Hyper-parameters can be optimised to increase the performance of classifiers, or they can be improved by categorising and expelling less related features [27].

## 3. Methodology

**Figure 1:**
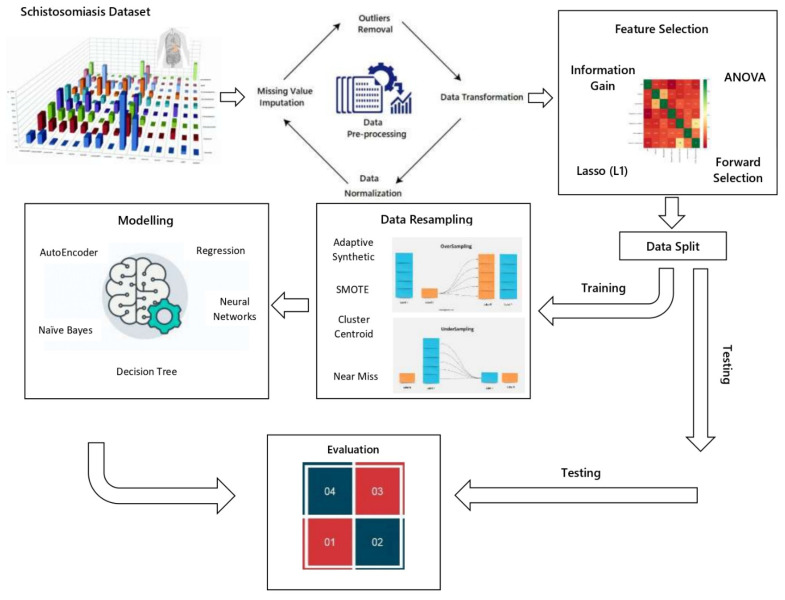
Proposed Methodology.

The detail of the proposed framework has been discussed in this section. Firstly, a dataset was obtained and then standardised to remove any biases among the various features. Then, several feature selection approaches are used to extract significant features from high-dimensional data [28,29]. In addition, several resampling approaches have been employed to address the data imbalance problem. After data preprocessing, various machine learning models were used, and the results were compared, as shown in Figure 1.

### 3.1. Dataset

A health record of Schistosomiasis has been analysed from the disease database compiled by the (HISPC) Hubei Institute of Schistosomiasis Prevention and Control, China. The patients with advanced Schistosomiasis gave information by surveying sociodemographic and epidemiological factors in Hubei. The (WS261–2006) National diagnostic criteria were used to assess Schistosomiasis. Advanced Schistosomiasis treatment varies from patient to patient depending upon disease conditions. If the patient’s condition is stable for six months, then praziquantel (PZD) treatment can be utilised. The dataset has been categorised into two groups containing 4136 individuals, both males and females. The 1232 cases in which improved patient health are referred to as favourable prognoses, coded as 0, while 2904 death cases are classified as poor prognoses, coded as 1. 18 features are recorded for each participant, as shown in Table 1 [1]. Data was gathered on socioeconomic, demographic, hospitalisation expenditures, clinical characteristics, and surgical procedures [30]. It was found that the area more exposed to water or lake containing marshy lands has a prevalence of Schistosomiasis, as the data also provides evidence.

### 3.2. Missing Data Imputation

An incomplete dataset for classification and prediction can reduce its effectiveness. The dataset containing large training data was more desirable for prediction because models would train better as more data was given to the model for training. The removal of missing values may increase the model’s performance. A dataset contains a high percentage of empty or blank cells, and it was unclear whether they should be replaced with zeros or remain as missing values. The methods for dealing with the imputation problem use mean attribute values from the original set [31]. The missing values in the dataset are replaced by the class’s median, as presented in Equations (1) and (2).

(1) $$\mathrm{For}\mathrm{odd}\mathrm{data}\mathrm{elements}=\frac{n+1}{2}\mathrm{th}\mathrm{term}$$

(2) $$\mathrm{For}\mathrm{even}\mathrm{data}\mathrm{elements}=\frac{\frac{n}{2}\mathrm{th}\mathrm{term}+\left(\frac{n}{2}+1\right)\mathrm{th}\mathrm{term}}{2}$$

### 3.3. Data Normalization

Data normalisation is a technique in which values are scaled and shifted to make new values [32]. We scaled the data ranging between 0 and 1, known as Z-score normalisation. Data normalisation is useful because all the features are in different forms or units. To normalise attributes in our dataset, Z-score normalisation has been used. It uses mean and standard deviation to normalise, as shown in Equation (3).

(3) $$X_{i}^{\prime }=\frac{X_{i}-\bar{E}}{\mathrm{std}(E)}$$

where $X_{i}^{\prime }$ Z-score is a normalised value and $X_{i}$ is the row E value of ith column or mean value. The $\mathrm{std}\left(E\right)$ and E are calculated using Equations (4) and (5).

(4) $$\mathrm{std}\left(E\right)=\sqrt{\frac{1}{\left(n-1\right)}\sum_{i=1}^{n}{{(X}_{i}-\bar{E})}^{2}}$$

(5) $$\bar{E}=\frac{1}{n}\sum_{i=1}^{n}X_{i}$$

### 3.4. Data Transformation

A process to convert or change data format to another format, structure, or value [33]. Our dataset contains the numerical features which have been converted into categorical values. After the literature survey, continuous features are converted to Boolean and continuous values [34,35], as shown in Table 1.

### 3.5. Feature Selection

An n-dimensional vector x is commonly used to represent data, with each element $x_{i}$ of x representing a feature. The dataset has multiple features, but not all of them will likely have a favourable effect on the target class [36]. Based on the input x and output y , the features using a scoring function $s\left(f\right)$ calculate filter scores where the feature is denoted by f. The filters are faster in processing and resistant to overfitting as compared to wrapper methods. Information gain [37], correlation-based technique [38], ReliefF [39], OneR [40], and gain ratio are the filters used in the scope of our study.

#### 3.5.1. Information Gain

The Information Gain (IG) approach is based on the information theory notion of entropy. This method ranks features (or input variables) according to the rate at which a variable reduces the target class’s uncertainty (entropy). Shannon’s entropy $E\left(s\right)$ measures how much uncertainty there is in a distribution and is calculated using Equation (6).

(6) $$E\left(s\right)=-\overset{m}{\underset{j=1}{\sum }}P\left(j\right)\log_{2}P$$

$P\left(j\right)$ is the probability for outcome j, and m is the number of alternative outcomes. To retrieve the information for all data with c number of classes, we employed Shannon’s entropy, as shown in Equation (7).

(7) $$\mathrm{Infogain}\left(C\right)=-\overset{c}{\underset{j=1}{\sum }}P_{c}{(b}_{i}{)\log}_{2}P_{c}\left(b_{i}\right)$$

$P_{c}\left(b_{i}\right)$ denotes the observed probability for each $P_{c}$ class. Data C has now been divided into L pieces $\left\{C_{1}{,C}_{2},\ldots {,C}_{L}\right\}$ by feature y . Equation (8) denoted the information for a portion $C_{k}$ .

(8) $$\mathrm{Info}\left(C_{k}^{y}\right)=-\overset{c}{\underset{j=1}{\sum }}P_{C_{k}^{y}}\left(b_{i}\right)\log_{2}P_{C_{k}^{y}}\left(b_{i}\right)$$

$\mathrm{Info}\left(C_{k}^{y}\right)=0$ if $P_{j}$ is zero then $\underset{P_{j}\to 0}{\lim}\log_{2}P_{j}=0$ . Due to feature y, the value of the information gain is calculated using Equation (9).

(9) $$\mathrm{InfoGain}\left(Y\right)=\mathrm{Infogain}\left(C\right)-\overset{m}{\underset{j=1}{\sum }}\frac{\left|C_{K}\right|}{\left|C\right|}\mathrm{Info}\left(C_{k}^{y}\right)$$

where $\left|C\right|$ and $\left|C_{K}\right|$ represent the number of instances in C and $C_{k}$ . Features with more distinct values are favoured by information gain. Considering Equation (9), more distinct characteristics do not affect the Infogain(C ), but they do have an impact on $-\overset{c}{\underset{j=1}{\sum }}P_{c}{(b}_{i}{)\log}_{2}P_{c}{(b}_{i})$ . For example, patient identification is a feature where each class has exactly one instance, with $C_{\mathrm{id}}=0$ for each patientId , maximising the amount of information gained. To put it another way, patientId is a good feature for the class but ineffective for generalisation. Quinlan et al. [41] proposed that information gain may be normalised using the information split technique, as presented in Equation (10).

(10) $$\mathrm{SplitInfogain}\left(Y\right)=-\overset{m}{\underset{j=1}{\sum }}\frac{\left|F_{j}\right|}{\left|F\right|}{*\log}_{2}\frac{\left|F_{j}\right|}{\left|F\right|}$$

F is the dataset, which is divided into $F_{j}$ datasets by feature Y. The information gain ratio is the normalised information gain measure, as shown in Equation (11).

(11) $$\mathrm{Gainratio}=\frac{\mathrm{InfoGain}(Y)}{\mathrm{SplitInfogain}(Y)}$$

#### 3.5.2. Correlation-Based Feature Selection (CFS)

The link between predictive factors and target variables, often called class, is the foundation of CFS. CFS identifies a selection of traits more correlated with the class than one another. As a result, CFS finds and removes redundant, irrelevant, and noisy features, leaving us with a subset of useful features. The single evaluation function is defined as in Equation (12).

(12) $${\mathrm{Eval}}_{A_{f}}=\frac{f\bar{b_{\mathrm{cf}}}}{\sqrt{f+f(f-1)\bar{b_{\mathrm{ff}}}}}$$

where ${\mathrm{Eval}}_{A_{f}}$ is the merit of a feature subset A with f features, $b_{\mathrm{cf}}$ is the average value of all feature-class correlations defined in Equation (13).

(13) $$\bar{b_{\mathrm{cf}}}=\frac{\sum_{j=1}^{f}b_{\mathrm{cfj}}}{F}$$

$b_{\mathrm{cfj}}$ indicates the correlation between feature j and the class, and $b_{\mathrm{ff}}$ is the average of feature correlations, with $b_{f_{j}f_{k}}$ indicating the correlation degree between features j and k as defined in Equations (14) and (15).

(14) $$\bar{b_{\mathrm{ff}}}=\frac{\sum_{j=1}^{f}\sum_{k=1}^{j-1}b_{f_{j}f_{k}}}{f_{p}}$$

(15) $$f_{p}=\frac{f\left(f-1\right)}{2}$$

$f_{p}$ is the amount of feature pairs in subset A, and an information-theoretic measure can be used to calculate the correlation matrix.


(16) $$b_{\mathrm{yz}}=\frac{\sum \mathrm{yz}}{m\sigma_{y}\sigma_{z}}$$

Here, m in Equation (16) shows instances of dataset whereas $\sigma_{x}\sigma_{z}$ shows the standard deviation of x and z, respectively. A weighted Pearson’s Correlation is calculated when one characteristic is linear, and the other is categorically shown in Equations (17) and (18).

(17) $$b_{yz}=\overset{w}{\underset{j=1}{\sum }}p\left(Y=y_{j}\right)b_{Y_{cj}Z}$$

(18) $$b_{\mathrm{YZ}}=\overset{w}{\underset{j=1}{\sum }}\overset{t}{\underset{k=1}{\sum }}{p(Y=y}_{j}{,Z=z}_{j}{))b}_{Y_{\mathrm{cj}}Z_{\mathrm{cj}}}$$

The likelihood that Y takes the jth value in the full training set is weighted in each of the estimated correlations.

#### 3.5.3. ReliefF

When dealing with real-time and noisy data, the reliefF algorithm is favourable. The reliefF algorithm selects the instances $s_{j}$ at random and then determines the k nearest neighbours in the similar and dissimilar classes [42]. These instances are referred to as $T_{i}$ , $R_{i}$ for nearest hit and miss. The Manhattan distance is commonly used to distinguish between $T_{i}$ and $R_{i}$ occurrences. Using $T_{i}$ , $s_{j}$ and $R_{i}$ for the updating, the quality estimation, $Q\left[E\right]$ . of all the attributes E is updated. If the values of the instances $T_{i}$ and $s_{j}$ are the same, the attribute E is divided into two instances with the same classes, which is necessary to reduce the $Q\left[E\right]$ and if values of both instances are different with E is divided into different classes of two instances then $Q\left[E\right]$ maximises. The entire procedure is performed n times, with n being a user-defined value. The $Q\left[E\right]$ is updated in this procedure by applying Equations (19)–(21).

(19) $$Q\left[E\right]=Q\left[E\right]+(\bar{T}+\bar{R})/n$$

(20) $$\bar{T}=-\overset{l}{\underset{i=1}{\sum }}{A(E,s}_{j}{,T}_{i})/l$$

(21) $$\bar{R}=\underset{B\neq {\mathrm{bl}(s}_{j})}{\sum }[(\frac{P(B)}{1-P(\mathrm{bl}\left(s_{j}\right))})\overset{l}{\underset{i=1}{\sum }}{A(E,s}_{j}{,R}_{i}(B))]/l$$

P(B) stands for the previous class, and A stands for the distance between the examples $s_{j}$ , B stands for classes and $\mathrm{bl}\left(s_{j}\right)$ stands for a class of jth sample. The eight most significant features that have been selected through various feature selection techniques are presented in Table 2, and the score values of all features are shown in Table 3.

### 3.6. Data Resampling

A dataset is imbalanced when the number of instances in one class does not equal other classes. The resampling methods are widely used to balance the dataset. Usually, the model trained on a balanced dataset achieves high results. Most medical-related datasets are imbalanced, which needs resampling approaches to balance the dataset, so multiple undersampling and oversampling techniques have been used in this study.

#### 3.6.1. Random Undersampling

In this approach, most class instances are randomly removed from the training set, so the ratio between both classes would be the same. The problem with this technique is that one would not know which information has been eliminated. The information which was very important for the study may be removed using Equation (22) [43].

(22) $$R\left(i\right)=\frac{S_{i}}{S}\forall i\in \left\{0,1\right\}$$

where $S_{i}$ represents the total number of values with the label i . Undersampling is denoted by $S_{0}^{\prime }$ and 0 means from class 0. ${R\left(0\right)}^{\prime \prime }R\left(1\right)$ Show that one class has more values than the other, and by combining both, we get Equation (23).

(23) $$\frac{R^{\prime }(0)}{R^{\prime }(1)}=\frac{\frac{S_{0}^{\prime }}{S^{\prime }}}{\frac{S_{1}}{S^{\prime }}}=1^{\prime \prime }\;\frac{R(0)}{R(1)}$$

When the dataset is rebalanced properly, this case can be applied $\left(\left(S_{0}^{\prime }=S_{1}\right)S_{1}\right)$ but when we have a scenario where the classes are balanced to a certain factor, inequality remains valid. On the contrary, when random points were selected for removal, the conditional data distribution between the classes was assumed to be unchanged, as shown in Equation (24).

(24) $$R^{\prime }\left(0\right)=R\left(0\right)$$

#### 3.6.2. Near Miss

Zhang and Mani [44] proposed a method to prevent information loss while utilising the undersampling technique. Instead of resampling the minority class, this strategy undersampling the majority class and making it equal to the minority class. This method uses the average distance between two points, a particular point, and the furthest points in the other class. NearMiss-1, 2, and 3 are the distinct versions. In NearMiss-1, we must choose a proportion of the major class size, and close to minor class points implies choosing the shortest average distance between the three minor class points. We chose the least average distance to the three farthest points from the minority class in NearMiss-2, which implies we chose the percentage of the majority class size closest to all points of the minority class. Select the nearest majority class points for each minor class in NearMiss-3. In this study, the NearMiss-1 method has been used.

#### 3.6.3. Cluster Centroid

Cluster Centroids were proposed to minimise the loss of information in the majority class [45]. By calculating the ratio between both classes, Cluster Centroids substitutes the majority of samples with clusters of centroids using the K-means method. The loss of information has been minimised by making groups based on similarity. The K-means algorithm was used to get the clusters when applied to the data based on the level of undersampling. Then, the majority of samples from the clusters were replaced by the cluster centroids, which contain a different representation of the majority class at the centre from K-Means.

#### 3.6.4. Synthetic Minority Oversampling Technique

Chawla et al. [46] introduced the SMOTE as it is one of the easier and most successful to address the problem of class imbalance. It creates new minority class instances synthetically rather than repeating duplicates of minority class instances [47]. Class overlapping is a critical aspect that makes learning a good classifier hypothesis for an unbalanced dataset difficult. It over-samples by using k nearest neighbours from minority samples, calculating the difference, multiplying the difference by a random number, and adding it to the feature vector, as shown in Equation (25).

(25) $$y_{\mathrm{new}}=y_{i}+\left(y_{i}^{\prime }-y_{i}\right)*\alpha$$

$y_{i}^{\prime }$ is K-nearest neighbour of $y_{i}$ and α € [0; 1] is a random number. Borderline-SMOTE is a further variant of SMOTE that solves its limitation. The cases closer to the borderline (i.e., majority class examples) are more difficult to categorise correctly. This approach prioritises these examples to increase oversampling performance. It is similar to SMOTE in that it calculates synthetic examples based on the class with minimum samples on the borderline. For example, borderline-SMOTE [48] prioritises locating samples on the class boundaries, i.e., borderline samples, and then oversampling them to improve prediction classification techniques are predicated on learning these boundaries during training. The algorithm is largely based on SMOTE.

1. For each sample in the minority class $y_{iƐ}S_{\min}$ . Get the collection of k-nearest neighbours, $S_{\mathrm{Knn}}$ .
2. Determine the number of nearest neighbours who are members of the majority class $\left|S_{\mathrm{Knn}}\cap S_{\mathrm{maj}}\right|$ for each $y_{i}.$ Select observations such that in Equation (26):
(26) $$\frac{n}{2}\leq |S_{\mathrm{Knn}}\cap S_{\mathrm{maj}}|<n$$
3. After that, the observations are run through the standard SMOTE method to generate synthetic points that solely include the minority and majority classes.

Clustering, filtering, and oversampling are the three steps in K mean SMOTE [49] proposed by Douzas et al. [50]. The input samples are grouped into k groups using k-means clustering, then choosing clusters for oversampling at the filtering stage, keeping the high proportion of samples from minority classes. The amount of synthetic samples to be generated is then dispersed, with more samples being assigned to clusters where minority samples are sparsely distributed then, to achieve the required ratio of samples SMOTE is used.

Each cluster’s proportion of minority and majority instances is used to identify clusters for oversampling. Oversampling is selected by default for any cluster containing at least 50% minority samples; by changing the imbalance ratio threshold, we can change the behaviour, a k-means SMOTE hyperparameter that defaults to 1. The ratio is defined as $\frac{\mathrm{majoritycount}+1}{\mathrm{minoritycount}+1}$ of imbalanced instances. Cluster selection becomes more selective, requiring a higher ratio of minority samples to be selected when there is a criterion for an imbalance ratio. Lowering the barrier, on the other hand, loosens the selection criteria, making it possible to choose clusters with a bigger majority share and assigned sample weights between 0 and 1. They are calculated as follows:

1. Euclidean distance was calculated for selected clusters (s), ignoring the majority class. The mean distance of each cluster is obtained by adding all non-diagonal entries of the distance matrix and dividing them with non-diagonal elements.
2. Divide the number of minority instances in each cluster by the average minority distance raised to the power of the number of features n to get density, i.e., $\mathrm{density}\left(s\right)=\frac{\mathrm{minoritycount}\left(s\right)}{\mathrm{averageMinorityDistance}{\left(s\right)}^{n}}$ .
3. To acquire a measure of sparsity, invert the density measure: $\mathrm{sparsity}\left(s\right)=\frac{1}{\mathrm{density}\left(s\right)}$ .
4. We can calculate the cluster’s sampling weight by dividing the cluster’s sparsity factor by the sum of all cluster’s sparsity factors.

**Figure 2:**
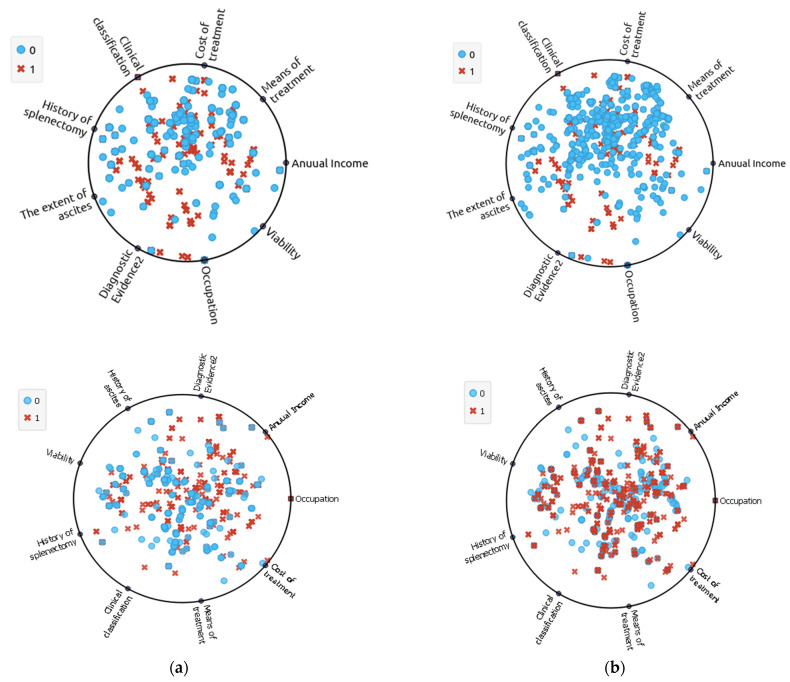
Visualisation of data before and after resampling: (**a**) Imbalanced dataset; (**b**) Balanced Dataset.

Resulting totalsamplingWeights=1 , i.e., $\left|\mathrm{samplingWeights}\left(s\right)*x\right|$ X is the total number of samples generated in that cluster that was oversampled using SMOTE. The hyperparameter k nearest neighbours, or KNN, in SMOTE, determines how many surrounding minority samples of $\vec{a}$ the point $\vec{b}$ are randomly chosen. When a cluster includes fewer than knn+1 minority samples, the value of KNN may need to be modified downward, depending on the SMOTE implementation. After SMOTE has been employed, both classes, minority and majority ones are the same. Figure 2 depicts the dataset before and after resampling. Table 4 presents the distribution of the dataset before and after resampling. 

### 3.7. Gradient Boosting

Gradient Boosting (GB) is an iterative machine learning-based approach for solving classification problems. This method is based on an ensemble learning model trained using the previous iteration’s mistakes as input. By fitting new learners, GB corrects misclassified data to the ensemble residual, which is the gap between the goal outputs and the ensemble’s current predictions. GB aims to maximise the ensemble’s prediction power while minimising bias. The benefit of Boosting is that it significantly impacts accuracy, but it comes at the expense of being slower to train because each learner is trained linearly.

It is a Boosting-like algorithm [51] and has a dataset $D_{t}={\left\{a_{i},b_{i}\right\}}_{1}^{M}$ for training, the goal of GB is to find an approximation, $\hat{A}$ , of the function $A^{*}\left(a\right)$ , which maps instances a to their output values b , by minimising the expected value of a given loss function, $Lf\left(b,A\left(a\right)\right)$ . GB builds an additive approximation of $A^{*}\left(a\right)$ as a weighted sum of functions calculated via Equation (27).

(27) $$A_{n}\left(a\right)=A_{n-1}\left(a\right){+w}_{n}g_{n}\left(a\right)$$

where $w_{n}$ is the weight of $n^{th}$ function and $g_{n}\left(a\right)$ are the parts of ensemble models. First, we obtain a constant approximation of $A^{*}\left(a\right)$ as shown in Equation (28).

(28) $$A_{0}\left(a\right)={\mathrm{argmin}}_{\beta }\overset{M}{\underset{i=1}{\sum }}{\mathrm{Lf}(b}_{i},\beta )$$

Models are minimised as in Equation (29). However, instead of solving the optimisation problem directly, each $g_{n}$ can be seen as a greedy step in a gradient descent optimisation for $A^{*}$ . For that, each model, $A^{*}$ is trained on a new dataset $D_{t}={\left\{a_{i},{\mathrm{pr}}_{\mathrm{ni}}\right\}}_{i=1}^{M}$ , where the pseudo-residuals ${\mathrm{pr}}_{\mathrm{ni}}$ , are calculated via Equation (29).

(29) $$(w_{n},\;g_{n}\left(a\right))={\mathrm{argmin}}_{w,g}\overset{M}{\underset{i=1}{\sum }}{\mathrm{Lf}(b}_{i}{,A}_{n-1}\left(a_{i}\right){+\mathrm{wg}(a}_{i}))$$

After that, a line search optimisation problem is used to calculate the value of $w_{n}.$ If the iterative procedure is not adequately regularised, this approach may suffer from over-fitting. If the model $g_{n}$ fully matches the pseudo-residuals for some loss functions (e.g., quadratic loss), the pseudo-residuals become zero in the following iteration, and the process finishes prematurely. Several regularisation hyper-parameters are examined to govern the additive process of GB. The natural way to regularise GB is to apply shrinkage to reduce each gradient descent step $A_{n}\left(a\right)=A_{n-1}\left(a\right)+w_{n}g_{n}\left(a\right)$ with $u=\left(0,1.0\right)$ . Typically, the value of u is set to 0.1. Furthermore, more regularisation may be obtained by reducing the complexity of the trained models. We can restrict the depth of decision trees or the minimum number of occurrences required to divide a node in the case of decision trees.

In contrast to a random forest, the default settings for these hyper-parameters in GB are designed to severely limit the expressive potential of the trees (for example, the depth is often limited to 3 to 5). Finally, various versions of GB incorporate hyper-parameters that randomise the base learners, such as random subsampling without substitution. These hyper-parameters can increase the generalisation of the ensemble [52]—Algorithm 1 lists all the steps of gradient boosting.
**Algorithms 1** Gradient Boosting
1.Initialising the constant

$A_{0}\left(a\right)={\mathrm{argmin}}_{\beta }\overset{M}{\underset{i=1}{\sum }}{\mathrm{Lf}(b}_{i},\beta )$


2.A For-Do loop (i=1→M)

3.At the training point, calculate the gradient. The new base learner was fitted to the target value to find the best gradient step. Then, update the estimate function

$(w_{n}{,g}_{n}\left(a\right))={\mathrm{argmin}}_{w,g}\overset{M}{\underset{i=1}{\sum }}{\mathrm{Lf}(b}_{i}{,A}_{n-1}\left(a_{i}\right){+\mathrm{wg}(a}_{i}))$

4.
i←i+1

5.Loop end
6.Return

XGBoost [53] is a highly scalable DT ensemble method based on GB. XGBoost, like GB, minimises a loss function because it is only interested in DT as a base classifier shown in Equation (30).

(30) $${\mathrm{Lf}}_{\mathrm{xgb}}=\overset{M}{\underset{i=1}{\sum }}{\mathrm{Lf}(b}_{i},A\left(a_{i}\right))+\overset{N}{\underset{n=1}{\sum }}Ω{(g}_{n})$$

(31) $$Ω\left(g\right)=\gamma \mathrm{Lt}+\frac{1}{2}{\pi v}^{2}$$

With the leaves of tree Lt in Equation (31), and the output scores v , the split criterion can be incorporated into decision trees’ loss function, resulting in a pre-pruning approach. In XGBoost, shrinkage is an additional regularisation hyper-parameter that reduces the step size in the additive expansion. Finally, other tactics, such as tree depth, limit tree complexity, reduce storage space and increase training speed. To train individual trees, random subsamples and column subsampling at the tree and tree node levels are among the randomisation approaches used in XGBoost. Furthermore, first and second-order gradients were obtained by constructing a function and sending it through the "objective" hyper-parameter.

XGBoost, in particular, focuses on minimising computation cost by determining the optimal split of DT building algorithms. In most split discovery algorithms, all feasible candidate splits are enumerated, and the One with the biggest gain has been chosen. A regular scan of each ranked attribute is required to discover the optimal split for each node. XGBoost employs a compressed column-based structure containing data in a pre-sorted form to prevent sorting the data in each node. XGBoost uses an approach based on data percentiles, in which just a sample of candidate splits is examined, and their gain is calculated using aggregated statistics. This study uses the parameters used to tune XGBoost: learning rate, minimum loss (gamma), max_dep, sample at each split, and subsampling rate.

Light Gradient Boosting (LGBoost) [54] executes GB and suggests several variations focusing on a computationally efficient approach, similar to XGBoost, based on histogram features and also provides hyper-parameters that allow being used in a range of scenarios: This works on both GPU and CPU, as simple GB, and has several options. Gradient-based One-Side Sampling (GOSS) and Exclusive Feature Bundling are two new features proposed by LGBoost. GOSS is a subsampling strategy for creating the training sets for the ensemble’s base trees. This strategy, like AdaBoost, tries to increase the relevance of cases, referred to as instances with a higher gradient.

The training consist of gradients $\left(x\right)$ and a sample fraction $\left(y\right)$ when the GOSS option is enabled, when computing the information gain, the instances are weighted by $\left(1-x\right)/y$ for the change in distribution. Many sparse features were combined with the Exclusive Feature Bundling (EFB) method into a single feature. GOSS and EFB both improve training speed. The parameters tuned for LGBoost are learning rate, max_num_leaves, highest_gradient, lowest_gradient, and feature_fraction_node.

#### CatBoost

CatBoost [55] is a GB library that reduces prediction shifts during training. This distribution shift is the difference between $A\left(a\right)|a$ with $a_{i}$ being a training instance and $A\left(a\right)|a$ for a test instance a . GB minimises gradients during training using the same instances for both estimates and models. CatBoost proposes that the gradients be estimated in their training set by a series of models which do not contain that occurrence. CatBoost does this by introducing a random permutation into the training cases. CatBoost’s logic is to create i=1,…,N base models for each of the M Boosting iterations. The gradient of the i+1 instance for the ( m+1)th Boosting iteration is estimated using the ith model of the mth iteration, trained on permutation first i instance. This method is repeated with j other random permutations in order to be independent of the first random permutation [56,57,58].

CatBoost is implemented so that a single model per iteration handles all permutations and models. Symmetric trees are formed by extending all leaf nodes level-wise with the same split condition to serve as the foundation models. The catBoost algorithm handles categorical features by replacing them with the numeric feature, which assesses the predicted goal value for each category. This numeric feature should preferably be generated using a different dataset to minimise overfitting the training data. This, however, is not always achievable. CatBoost proposes a strategy for computing this additional feature identical to the One used to generate the models. That is the information of instances<i is utilised to determine the feature value of instance i for a given random permutation of the instances. The feature value acquired for each instance is then averaged after many variations. Computing target statistics for categorical features, like EFB in the LGBoost model, is a preprocessing approach. CatBoost is a large library with many features like GPU training, different parameters according to scenarios, and Boosting standards.

## 4. Results

The model has been trained on training data and then uses the test set to assess the model’s generalisation with a 70:30 split approach. When evaluating the performance of classifiers, performance assessment is crucial. A confusion matrix has been used to evaluate the performance of a classifier. The confusion matrix is a multidimensional table that depicts successfully or incorrectly predicted times for each class in real and projected costs for all times. Any predictive modelling assignment necessitates the evaluation of models. It is considerably more important in predictive modelling, whereas the performance and variety of classifiers must be assessed comprehensively. Each of the assessment measures is based on one of four classes: true positive (TP), true negative (TN), false positive (FP), and false negative (FN). The experiments and results show that our model achieved state-of-the-art performance. We evaluate our model by comparing it with other machine-learning models. The results are compared in terms of accuracy, precision, recall, and F1-Score, which can be calculated using parameters which are defined as [59,60,61,62]:

- **True Positive (TP):** It identifies how much the data instances are identified as recovery cases.
- **True Negative (TN):** It identifies how many data instances are categorised as death cases.
- **False Positive (FP):** It identifies how much the data instances are incorrectly categorised as recovery cases.
- **False Negative (FN):** It Identifies how many data instances are incorrectly categorised as death cases;

**Accuracy** is the ratio of all correctly predicted samples to the summation of total predictions, as shown in Equation (32).

(32) $$\mathrm{Accuracy}=\frac{\mathrm{TP}+\mathrm{TN}}{\mathrm{TP}+\mathrm{TN}+\mathrm{FP}+\mathrm{FN}}$$

**Precision:** It identifies if the positive predictions are correctly determined and is the ratio of TP to the summation of TP and FP, as shown in Equation (33).

(33) $$\mathrm{Precision}=\frac{\mathrm{TP}}{\mathrm{TP}+\mathrm{FP}}$$

**Recall:** It identifies total relevant results correctly predicted by the model and is the ratio of TP to the summation of TP and FN, as shown in Equation (34).

(34) $$\mathrm{Recall}=\frac{\mathrm{TP}}{\mathrm{TP}+\mathrm{FN}}$$

**F1-Score:** It is characterised as the harmonic mean of the model’s precision and recall and is a way to integrate the model’s precision and recall. It is stated in Equation (35).

(35) $$F1\text{-}\mathrm{Score}=2\times \frac{\mathrm{Precision}\times \mathrm{Recall}}{\mathrm{Precision}+\mathrm{Recall}}$$

Before resampling, different feature selection techniques have also been used to reduce the data dimensionality. We have applied the five feature selection techniques: Correlation, Information gain, Gain ratio, ReliefF and OneR. We have selected eight features by using these feature selection techniques. Each feature selection approach scores all features; hence each technique ranks the features. Which enables the selection of the most important characteristics k using a feature selection approach. ∀j∈J where J be a set contain integers and (j ∈ [1, 2, …, n − 1, n]. Each j specifies the amount of the experiment’s best features that must be assessed. In the best-case scenario, J includes all numbers in the range [1, 2, …, n − 1, n]. Because feature selection strives to choose the best features with respect to scorer. The feature selection technique’s assessment depends on the score of the underlying feature selection approach, and for each method, an experiment was run numerous times. Furthermore, we utilised undersampling and oversampling techniques to balance the dataset.

The GB algorithm we used with the combination of hyperparameter tuning, which are learning-rate = 0.1, the number of tress (n-estimators = 100), max-depth = 3, min-samples-split = 2, min-sample-leaf = 1 and subsample = 1. The combination of the parameter used by LGBoost is max-depth = 20, num-of–iterations or estimator = 100, num-leaves = 31, and sub-sample = 1. The other parameter, like L1, and L2 regularisation or the alpha value, was 0. For XGBoost, we have set the parameter’s value as learning rate = 0.1, n-estimator = 100, subsample = 1 and max-depth = 3. When tuning XGBoost, we have to take care of mostly 3 parameters: number-of-tree, trees-depth and stepsize to achieve better results. The parameters used for the CatBoost algorithm are: iteration = 1000, leaf-estimation-iterations = 100, depth = 7, L2 regularisation = 5, learning-rate = 0.03, and other parameters like bagging-temperature, random-strength, leaf-estimation, eval-metric, bootstrap-type and loss-function are optional. As our model was a binary class, parameters like loss-function and eval-metric were not mentioned, but in the case of multiclass need to mention it. Table 5 shows the result of the random undersampling technique with multiple feature selection techniques. The GB model performed poorly, with an accuracy of 68.1% in the case of ReliefF and 68.6% with the OneR feature selections technique [63,64,65]. The XGBOOST model performed very well for correlation and information gain feature selection techniques with an accuracy of 82.8%. In contrast, the CatBoost model performed very well for the Gain ratio feature selection technique leading in the case of recall with a value of 82.7%.

**Figure 3:**
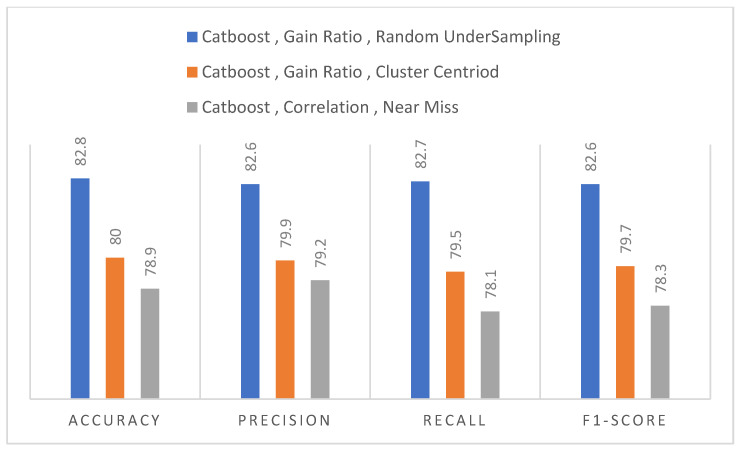
Result of CatBoost models to feature selection undersampling technique.

Table 6 and Table 7 show the results of Cluster Centroid and Near Miss techniques. The GB model performed well in the case of the Gain ratio with an accuracy of 78.9%, shown in Table 6, and the CatBoost model performed well overall in both tables with an accuracy of 80% and 78.9%. Still, the results obtained from both undersampling techniques were less than the previous study done by Li G et al. [1]. The CatBoost performed very well classifying both classes when undersampling techniques were used. However, in the case of the undersampling, the accuracy decreased to 78.9% when clustering centroid techniques were used and 79.2 for Near miss, which was poor because, on an imbalanced dataset, Li G et al. [1] achieved an accuracy of 80%. Even though the accuracy achieved by CatBoost using random undersampling techniques was 82.7%, greater than Li G et al. [1], we cannot neglect oversampling techniques. As in Figure 3, a graph plot shows the highest result to feature selection and resampling techniques.

Table 8 contains the result of oversampling techniques to feature selection techniques. Table 8 shows the result of the random oversampling technique for multiple feature selection techniques. All OneR and ReliefF models performed very poorly, with the highest accuracy of 68.3% and 73.3%, whereas they performed well in the Gain ratio with an accuracy of 82.9%. Both XGBoost and CatBoost models obtained the highest result of 82.9 % concerning correlation and Information gain feature selection techniques.

Table 9 shows the outcome of the SMOTE oversampling approach in comparison to numerous feature selection strategies. In terms of multiple feature selection strategies, the results produced using these resampling techniques have been less than those obtained using random oversampling techniques. In the OneR and ReliefF tests, models concerning those parameters underperformed with values of 75.9% and 71.0% but well in the Gain Ratio test with a value of 82.9 by LGBoost. Regarding correlation and information gain feature selection strategies, both the XGBoost and CatBoost models came on top, but XGBoost took the lead with a value of 83.3%.

Table 10 presents the results of an oversampling strategy K-means SMOTE and various feature selection procedures. The result obtained using this resampling approach was the best of all the resampling techniques in terms of multiple feature selection strategies. Utilising this resampling method, the results obtained from models employing feature selection techniques, Information gain, ReliefF, and OneR were unsatisfactory; however, models using Gain ratio and Correlation feature selection strategies did extremely well with a value of 87.1%. In addition, the CatBoost model outperformed other models.

**Figure 4:**
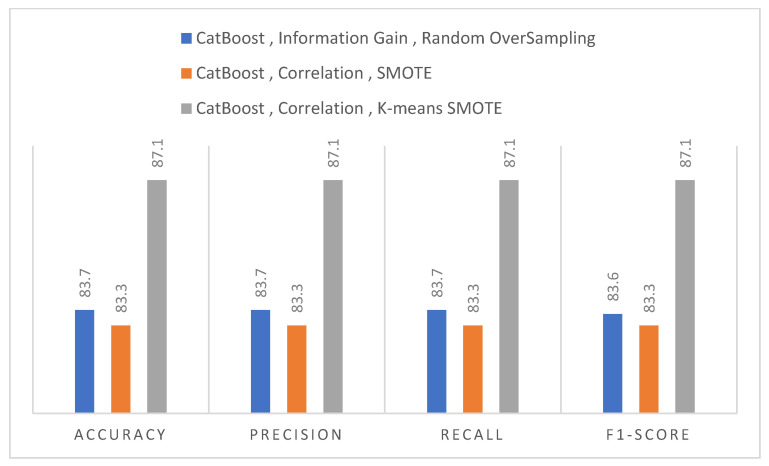
Result of CatBoost models to Oversampling and feature selection technique.

Summarising the K means SMOTE approach when utilised, the results were more efficient when using the correlation-based feature selection technique. The table, as shown above, summarises the performance evaluation of several classifiers using a selected feature subset from each feature selection approach and method. The CatBoost approach’s performance was also compared to that of typical machine learning models, and it was found that the CatBoost method outperformed traditional machine learning models. Table 5 shows the findings, which show that the CatBoost model produced the greatest results, with an accuracy of 87.1 percent, also shown in Figure 4.

## 5. Discussion

Machine learning and data mining approaches can automatically discover complex patterns in the healthcare domain, such as COVID-19 [66,67], Skin Cancer [59], Breast Cancer [16], Malignant Mesothelioma [68,69,70], and Cervical Cancer [7], researchers are motivated to use these techniques in the early prediction of Schistosomiasis. In recent years, many machine learning methods, including LR, DT, RF, and ANN, have been widely applied in disease detection and prediction [59]. Before resampling, different feature selection techniques have also been used to reduce the data dimensionality. We have applied the five feature selection techniques: Correlation, Information gain, Gain ratio, ReliefF and OneR. We have selected eight features by performing multiple experiments, as explained above. Furthermore, we utilised undersampling and oversampling techniques to balance the dataset. We have used four algorithms, GB, LGBoost, XGBoost and CatBoost, with a combination of various hyperparameters discussed in the result section.

The results of the random undersampling approach with several feature selection strategies are shown in Table 5. The GB model fared badly, with an accuracy of 68.1% for ReliefF and 68.6% for OneR feature choices. The XGBOOST model fared extremely well for correlation and information gain feature selection approaches, with an accuracy of 82.8%, while the CatBoost model did very well for the Gain ratio feature selection technique, with an accuracy of 82.7%. The outcomes of the Cluster Centroid and Near Miss approaches are shown in Table 6 and Table 7. The GB model fared well in the Gain ratio instance, with an accuracy of 78.9% in Table 6, while the CatBoost model did well overall in both tables, with an accuracy of 80% and 78.9%.

Nonetheless, the findings from both undersampling strategies were lower than the prior study by Li G et al. [1]. When undersampling techniques were utilised, the CatBoost performed admirably in categorising both groups. However, when undersampling was utilised, the accuracy dropped to 78.9% when clustering centroid approaches were applied, and 79.2% for Near miss, which was disappointing considering Li G et al. [1] obtained an accuracy of 80% on an unbalanced dataset. Even though CatBoost’s accuracy utilising random undersampling strategies was 82.7%, which was higher than Li G et al. [1] as shown in a graph plot, Figure 3, displays the best outcome for feature selection and resampling procedures.

The results of the random oversampling approach for different feature selection strategies are shown in Table 8. All models fared badly with OneR and ReliefF techniques, with the greatest accuracy of 68.3% and 73.3%, respectively. However, they did well in the Gain ratio, with an accuracy of 82.9%. In terms of correlation and information gain feature selection strategies, both the XGBoost and CatBoost models achieved 82.9%. Table 9 compares the SMOTE oversampling strategy to various feature selection algorithms. The results achieved via these resampling procedures were lower than those acquired by random oversampling techniques. Models considering such parameters underperformed at 71.0% in the OneR and ReliefF tests. XGBoost takes the lead in correlation and information gain feature selection algorithms, with a value of 83.3%. Table 10 shows the outcomes of a K-means SMOTE oversampling technique and several feature selection procedures. In terms of various feature selection strategies, the result of this resampling methodology was the best of all resampling procedures. Using this resampling strategy, the results from models utilising feature selection procedures, Information gain, ReliefF, and OneR were poor; however, models adopting Gain ratio and Correlation feature selection strategies performed very well, with a value of 87.1%. Other models were outperformed by the CatBoost model. When the K-means SMOTE strategy was applied, the results were more efficient with the correlation-based feature selection technique. Table 10 summarises the performance evaluation of classifiers. The performance of the CatBoost technique was also compared to that of standard machine learning models, and it was discovered that the CatBoost method beat traditional machine learning models. The outcomes are provided in Table 10, which reveals that the CatBoost model gave the best results, with an accuracy of 87.1 %, as shown in Figure 4.

CatBoost was more accurate than other methods in our research on different medical conditions. In the CatBoost model, we discovered that ascitic fluid volume was the most useful predictor. The most prevalent sign of advanced hepatic illness is ascites. The ascitic subtype of advanced Schistosomiasis is the most dangerous, accounting for 65–90 percent of cases [60]. Granulomatous inflammation can be caused by venous blockage and portal hypertension and leads to continuous fibrosis and a drop in plasma colloid osmotic pressure [61]. Ascites are the most common consequence of liver injury, and their severity directly influences the overall prognosis. In advanced Schistosomiasis patients, the occurrence of severe ascites is one of the best indicators of a high level of impairment [62]. This study has demonstrated that CatBoost is a novel and promising modelling framework for the unfavourable prognosis of advanced Schistosomiasis. A comparison between our model and the previous studies is shown in Table 11.

## 6. Conclusions

Machine learning methods have been used in various fields in combination with unbalanced approaches. This study aims to use supervised learning algorithms to train multiple Schistosomiasis disease prediction systems. Because an unbalanced dataset is important for improving the model’s performance in classification challenges, several resampling strategies were utilised to balance the dataset. We began by employing exploratory data analysis approaches, such as data standardisation, to analyse the dataset. Then, we conducted several tests to validate the performance of feature selection strategies against one another and narrow down the characteristics that may properly diagnose the disease. These variables can be addressed early in the disease to the point where it threatens human life. We began by comparing the outcomes of several GB models, such as LGBoost, XGBoost, and CatBoost, against each other and earlier studies. The CatBoost model has a greater prediction accuracy rate than typical machine learning-based models. Using the K-means SMOTE resampling approach, the CatBoost method had the greatest accuracy of 87.1%. We will utilise more datasets linked to this disease in the future to include the features categorised as important by the dataset collector but not included in this dataset. Finally, we will release this framework on the web so medical professionals can take benefit from it.

## Figures and Tables

**Table 1:** Data Transformation.

Name	Description			Range
Occupation	- Farmer - Other			Categorical
Annual Income	- Less than 10,000 = 0 - Equal or greater than 10,000 = 1			Boolean
Height Weight	BMI	>18.5—Underweight	0	Categorical
		18.5–24.5 Normal	1	
		25.0–29.5 overweight	2	
		≤30.0 Obese	3	
Viability/Development (disease)	- Normal = 1 - Poor = 2 - Supernormal =3			Categorical
Nourishment (nutrient level inside the body)	- Well =1 - Medium = 2 - Bad = 3 - Cachexia/severe = 4			Categorical
Diagnostic Evidence 1	- Blood Test = 1 - Stool test = 2 - Blood + stool = 3 - Proctoscopy = 4			Categorical
Diagnostic Evidence 2	- Ascites (Grade 1, 2, 3) = 1–3 - Non-Ascites = 4			Categorical
Prior Treatment	- Yes =1 - No = 0			Boolean
History of Splenectomy	- Yes = 1 - No = 0			Boolean
History of ascites	- Yes = 1 - No = 0			Boolean
Other Disease	- No = 0 - Cardiovascular = 1 - Digestive = 2 - Neuropsychiatric = 3 - Respiratory = 4 - Urinary = 5 - Other = 6 - Cardiovascular + Digestive = 12 - Digestive + Urinary = 25 - Digestive + other = 26 - Urinary + other = 56			Categorical
Extent of Ascites	- Mild to moderate = 0 - Severe = 1			Boolean
Clinical Classification	- Ascites = 1 - Megalosplenia = 2 - Colonic tumoroid proliferation = 3 - Dwarfism and others = 4			Categorical
Type of treatment the patient	- New patient (Schistosomiasis newly diagnosed) - Retreated patient (Improved after treatment and will be treated again next year) - Relapsed patient (clinically cured but need further treatment for disease recurrence)			Categorical
Means of Treatment	- Medical - Surgical - Medical + Surgical			Categorical
Cost of Treatment	- Less than 5876.9 yuan - Greater or equal to 5876.9 yuan			Categorical

**Table 2:** Selected features from multiple feature selection techniques.

OneR	Information Gain	ReliefF	Gain Ratio	Correlation
Viability	Cost of treatment	Diagnostic Evidence 1	The extent of ascites	Cost of treatment
Means of treatment	Annual Income	BMI	Viability	Annual Income
The extent of ascites	Clinical classification	Clinical classification	Clinical classification	Clinical classification
BMI	History of splenectomy	Type of treating patients	Means of treatment	History of splenectomy
Diagnostic Evidence 2	Viability	History of ascites	Cost of treatment	Viability
Diagnostic Evidence 1	The extent of ascites	Diagnostic Evidence 2	Annual Income	Means of treatment
Nourishment	Means of treatment	Cost of treatment	History of splenectomy	The extent of ascites
Annual Income	Diagnostic Evidence 1	History of splenectomy	Occupation	Diagnostic Evidence 2

**Table 3:** Features Score.

Features	Score
Cost of treatment	0.07295
Annual Income	0.04749
Clinical classification	0.00604
History of splenectomy	−0.05814
Viability	−0.05815
Means of treatment	−0.06133
The extent of ascites	−0.07251
Diagnostic Evidence 2	−0.07253
Diagnostic Evidence 1	−0.07254
Type of treating patients	−0.07434
Occupation	−0.11227
BMI	−0.11531
Nourishment	−0.16426
History of ascites	−0.17023
Prior treatment	−0.20311
Other diseases	−0.27979

**Table 4:** Dataset Resampling.

Total Instances	Status	Imbalanced Dataset Instance	After Undersampling	After Oversampling
4136	Recovery	1232	1232	2904
	Death	2904	1232	2904

**Table 5:** Random undersampling to each feature selection technique.

Performance	Classifier	Correlation	Info Gain	ReliefF	OneR	Gain Ratio
Accuracy (%)	GB	82.2	82.7	**68.1**	**68.6**	82.2
	LGBoost	82.2	82.2	67.4	64.8	81.8
	XGBoost	**82.8**	**82.8**	67	66.4	82.4
	CatBoost	82.7	82.7	67.7	66.4	**82.8**
Precision (%)	GB	82.5	82.5	**67.7**	**68.6**	82.1
	LGBoost	82.1	82.1	67.1	66.3	81.1
	XGBoost	**82.6**	**82.6**	66.6	67.4	82.2
	CatBoost	82.5	82.5	67.4	67.1	**82.6**
Recall (%)	GB	82.5	82.5	**67.4**	**68.8**	82.1
	LGBoost	82.1	82.1	66.6	65.9	81.8
	XGBoost	**82.6**	**82.6**	66.3	67.2	82.3
	CatBoost	82.5	82.5	66.7	67.1	**82.7**
F1-Score (%)	GB	82.5	82.5	**67.5**	**68.5**	82.1
	LGBoost	82.1	82.1	66.8	64.7	81.7
	XGBoost	**82.6**	**82.6**	66.4	66.4	82.2
	CatBoost	82.5	82.4	66.8	66.4	**82.6**

**Table 6:** Results of Cluster Centroid to each feature selection technique.

Performance	Classifier	Correlation	Info gain	ReliefF	OneR	Gain Ratio
Accuracy (%)	GB	76.4	74.8	68.5	64.1	78.9
	LGBoost	78.1	77.9	68.7	63.6	78.6
	XGBoost	78.5	77.9	67.9	63.9	79.4
	CatBoost	**78.7**	**78.1**	**69**	**64.7**	**80**
Precision (%)	GB	76.3	74.6	68.2	63.7	78.7
	LGBoost	77.9	77.7	68.5	63.9	78.4
	XGBoost	78.4	77.7	67.6	64.2	79.3
	CatBoost	**78.7**	**77.9**	**68.7**	**65**	**79.9**
Recall (%)	GB	76.4	74.4	68.3	63.4	78.4
	LGBoost	77.7	77.9	68.6	64	78.3
	XGBoost	78	77.9	67.6	64.3	79.1
	CatBoost	**78.3**	**78**	**68.8**	**65.1**	**79.5**
F1-Score (%)	GB	76.3	74.5	68.3	63.4	78.7
	LGBoost	77.8	77.8	68.5	63.6	78.4
	XGBoost	78.1	77.8	67.7	63.9	79.1
	CatBoost	**78.4**	**77.9.**	**68.8**	**64.7**	**79.7**

**Table 7:** Results of Near Miss with respect to each feature selection technique.

Performance	Classifier	Correlation	Info Gain	ReliefF	OneR	Gain Ratio
Accuracy (%)	GB	78.6	78.6	64.5	71.8	**78.6**
	LGBoost	78.1	78.1	68.3	72.1	77.5
	XGBoost	78.7	78.7	68.2	72.4	78.1
	CatBoost	**78.9**	**78.9**	**68.6**	**71.8**	78.1
Precision (%)	GB	78.9	78.9	66.7	71.7	**78.9**
	LGBoost	78.1	78.1	68.3	72.1	77.7
	XGBoost	79.1	79.1	68.1	72.4	78.3
	CatBoost	**79.2**	**79.2**	**68.5**	**71.7**	78.3
Recall (%)	GB	77.8	77.8	65.9	71.9	**77.8**
	LGBoost	77.4	77.4	68.4	72.4	76.8
	XGBoost	77.8	77.9	68.3	**72.6**	77.3
	CatBoost	**78.1**	**78.1**	**68.7**	71.9	77.3
F1-Score (%)	GB	78.1	78.1	64.4	71.7	**78.1**
	LGBoost	77.6	77.6	68.2	72.1	77
	XGBoost	78.2	78.2	68.1	**72.3**	77.5
	CatBoost	**78.3**	**78.3**	68.5	71.7	77.5

**Table 8:** Results of models with respect to Random Oversampling and feature selection technique.

Performance	Classifier	Correlation	Info Gain	ReliefF	OneR	Gain Ratio
Accuracy (%)	GB	82.6	82.6	71.6	68	82
	LGBoost	83.4	83.4	**73.3**	67.6	82.9
	XGBoost	83.4	83.4	73	**68.3**	82.9
	CatBoost	**83.7**	**83.7**	72.9	67.8	**82.9**
Precision (%)	GB	82.6	82.6	72.1	68	82
	LGBoost	83.4	83.4	74.1	67.9	82.9
	XGBoost	83.5	83.5	**73.6**	**68.4**	82.9
	CatBoost	**83.7**	**83.7**	73.1	67.9	**82.9**
Recall (%)	GB	82.6	82.6	71.5	68	82
	LGBoost	83.4	83.4	**73.2**	67.7	82.9
	XGBoost	83.4	83.4	73	**68.3**	82.8
	CatBoost	**83.7**	**83.7**	72.9	67.8	**82.9**
F1-Score (%)	GB	82.6	82.6	71.4	68	82
	LGBoost	83.4	83.4	**73**	67.5	82.9
	XGBoost	83.4	83.4	72.8	**68.2**	82.8
	CatBoost	**83.6**	**83.6**	72.8	67.8	**82.9**

**Table 9:** Results of the models with respect to SMOTE and feature selection techniques.

Performance	Classifier	Correlation	Info Gain	ReliefF	OneR	Gain Ratio
Accuracy (%)	GB	82.4	82.4	74.8	69.7	82.2
	LGBoost	83.1	83.1	76.1	70.9	**82..9**
	XGBoost	**83.3**	**83.3**	75.8	70.7	82.8
	CatBoost	82.9	82.9	**75.9**	**71.0**	82.7
Precision (%)	GB	82.4	82.4	75.0	69.7	82.2
	LGBoost	83.2	83.2	**76.4**	71.5	**82.9**
	XGBoost	**83.3**	**83.3**	76.1	71.3	82.4
	CatBoost	82.9	82.9	76.2	**71.8**	82.7
Recall (%)	GB	82.4	82.4	74.7	69.7	82.2
	LGBoost	83.1	83.1	**76.1**	70.9	**82.9**
	XGBoost	**83.3**	**83.3**	75.8	70.8	82.8
	CatBoost	82.9	82.9	75.8	**71.1**	82.7
F1-Score (%)	GB	82.4	82.4	74.7	69.7	82.2
	LGBoost	83.1	83.1	**76.0**	70.7	**82.9**
	XGBoost	**83.3**	**83.3**	75.7	70.6	82.8
	CatBoost	82.9	82.9	75.8	**70.8**	82.7

**Table 10:** Results of the models with respect to K means SMOTE and feature selection techniques.

Performance	Classifier	Correlation	Info Gain	ReliefF	OneR	Gain Ratio
Accuracy (%)	GB	86.0	76.9	74.8	77.6	86.2
	LGBoost	86.7	**79.2**	76.1	77.9	86.4
	XGBoost	86.9	79.1	76.0	78.0	86.6
	CatBoost	**87.1**	79.1	**77.0**	**78.4**	**86.8**
Precision (%)	GB	86.0	77.0	74.8	78.0	86.4
	LGBoost	87.0	**79.3**	76.8	77.9	86.6
	XGBoost	87.2	79.1	76.6	78.0	86.9
	CatBoost	**87.1**	79.3	**77.7**	**78.4**	**87.0**
Recall (%)	GB	85.9	77.0	74.8	77.6	86.2
	LGBoost	86.7	**79.2**	76.1	77.9	86.3
	XGBoost	86.9	79.1	76.0	78.0	86.6
	CatBoost	**87.1**	79.1	**77.0**	**78.4**	**86.7**
F1-Score (%)	GB	85.9	76.9	74.8	77.5	86.2
	LGBoost	86.7	**79.2**	75.9	77.9	86.3
	XGBoost	86.9	79.1	75.9	78.0	86.6
	CatBoost	**87.1**	79.1	**76.8**	**78.4**	**86.7**

**Table 11:** Comparison of previous studies.

Reference	Year	Objective	Approach	Undersampling Technique	Oversampling Technique	Outcome
[27]	2017	To check the pattern analysis of Schistosomiasisdisease	K-mean algorithm, clustering	✗	✗	N.A
[1]	2018	To detect advanced Schistosomiasis in the people of Hubei province	ANN, DT, LR	✗	✗	80%
[21]	2019	To detect and predict the diagnosis of disease on an imbalanced dataset	PNN, RF, one rule, and DT	✗	✗	82%
[28]	2019	Detection of disease factor	Association Rule Mining	✗	✗	N.A
[29]	2019	Relationship between climate change and disease factor	K- means clustering	✗	✗	N.A
[18]	2020	Comparison to check the level of susceptibility of Schistosomiasis	SVM, Gaussian Methods	✗	✗	76.6–94%
[17]	2021	1 year prognosis for advanced Schistosomiasis	LR, RF, DT, ANN, XGBoost	✗	✗	79%
[30]	2021	Identify the high-risk areas of Schistosomiasis	LR, RF, GB	✗	✗	73–87%
**Our study**	**2022**	**Early prediction of Schistosomiasis**	**GB, LGBoost, XGBoost, CatBoost**	**✓**	**✓**	**87.1%**

## Data Availability

The data will be available upon request.

## References

[1] Li G., Zhou X., Liu J., Chen Y., Zhang H., Chen Y., Liu J., Jiang H., Yang J., Nie S. (2018). Comparison of three data mining models for prediction of advanced schistosomiasis prognosis in the Hubei province. PLoS Negl. Trop. Dis..

[2] Fusco T., Bi Y., Wang H., Browne F. (2020). Data mining and machine learning approaches for prediction modelling of schistosomiasis disease vectors: Epidemic disease prediction modelling. Int. J. Mach. Learn. Cybern..

[3] Olveda D.U., Olveda R.M., Jan Montes C., Chy D., Modesto Abellera III J.B., Cuajunco D., Lam A.K., McManus D.P., Li Y., Ross A.G. (2014). Clinical management of advanced Schistosomiasis: A case of portal vein thrombosis-induced splenomegaly requiring surgery. Case Rep..

[4] Huang L.H., Qiu Y.W., Hua H.Y., Niu X.H., Wu P.F., Wu H.Y., Zhu H.Y., Yang X.J., Yao S.Z., Li Y.G. (2013). The efficacy and safety of entecavir in patients with advanced Schistosomiasis co-infected with hepatitis B virus. Int. J. Infect. Dis..

[5] Schistosomiasis (Bilharzia). https://www.who.int/health-topics/schistosomiasis#tab=tab_1.

[6] Zhang L.J., Xu Z.M., Dang H., Li Y.L., Lü S., Xu J., Li S.Z., Zhou X.N. (2020). Endemic status of schistosomiasis in People’s Republic of China in 2019. Zhongguo Xue Xi Chong Bing Fang Zhi Za Zhi.

[7] Alam T.M., Milhan M., Khan A., Iqbal M.A., Wahab A., Mushtaq M. (2019). Cervical Cancer Prediction through Different Screening Methods Using Data Mining. IJACSA Int. J. Adv. Comput. Sci. Appl..

[8] Osakunor D.N.M., Woolhouse M.E.J., Mutapi F. (2018). Paediatric schistosomiasis: What we know and what we need to know. PLoS Negl. Trop. Dis..

[9] Ashour A.S., Hawas A.R., Guo Y. (2018). Comparative study of multiclass classification methods on light microscopic images for hepatic schistosomiasis fibrosis diagnosis. Health Inf. Sci. Syst..

[10] Zhao M., Zhang Z., Chow T.W.S. (2012). Trace ratio criterion based generalised discriminative learning for semi-supervised dimensionality reduction. Pattern Recognit..

[11] Baig T.I., Khan Y.D., Alam T.M., Biswal B., Aljuaid H., Gillani D.Q. (2022). ILipo-PseAAC: Identification of lipoylation sites using statistical moments and general PseAAC. Comput. Mater. Contin..

[12] Tariq A., Awan M.J., Alshudukhi J., Alam T.M., Alhamazani K.T., Meraf Z. (2022). Software Measurement by Using Artificial Intelligence. J. Nanomater..

[13] Alam T.K., Shaukat M., Mushtaq M., Ali Y., Khushi M. Corporate Bankruptcy Prediction: An Approach Towards Better Corporate World. https://academic.oup.com/comjnl/article-abstract/64/11/1731/5856206.

[14] Alam T.K., Shaukat I., Hameed S., Li J., Khushi M. An Investigation of Credit Card Default Prediction in the Imbalanced Datasets. https://ieeexplore.ieee.org/abstract/document/9239944/.

[15] Baig T.I., Alam T.M., Anjum T., Naseer S., Wahab A., Imtiaz M., Raza M.M. Classification of Human Face: Asian and Non-Asian People. Proceedings of the 2019 International Conference on Innovative Computing (ICIC).

[16] Ghani M.U., Alam T.M., Jaskani F.H. Comparison of Classification Models for Early Prediction of Breast Cancer. Proceedings of the 2019 International Conference on Innovative Computing (ICIC).

[17] Jiang H., Deng W., Zhou J., Ren G., Cai X., Li S., Hu B., Li C., Shi Y., Zhang N. (2021). Machine learning algorithms to predict the 1 year unfavourable prognosis for advanced Schistosomiasis. Int. J. Parasitol..

[18] Olanloye O.D., Olasunkanmi O., Oduntan O.E. (2020). Comparison of Support Vector Machine Models in the Classification of Susceptibility to Schistosomiasis. Balk. J. Electr. Comput. Eng..

[19] Asarnow D., Singh R. Determining Dose-Response Characteristics of Molecular Perturbations in Whole-Organism Assays Using Biological Imaging and Machine Learning. Proceedings of the 2018 IEEE International Conference on Bioinformatics and Biomedicine (BIBM).

[20] Kasse B., Gueye B., Diallo M., Santatra F., Elbiaze H. IoT based Schistosomiasis Monitoring for More Efficient Disease Prediction and Control Model. Proceedings of the 2019 IEEE Sensors Applications Symposium (SAS).

[21] Chicco D., Rovelli C. (2019). Computational prediction of diagnosis and feature selection on mesothelioma patient health records. PLoS ONE.

[22] He H., Garcia E.A. (2009). Learning from imbalanced data. IEEE Trans. Knowl. Data Eng..

[23] Chawla N.V., Bowyer K.W., Hall L.O., Kegelmeyer W.P. (2002). SMOTE: Synthetic Minority Over-sampling Technique. J. Artif. Intell. Res..

[24] Mazurowski M.A., Habas P.A., Zurada J.M., Lo J.Y., Baker J.A., Tourassi G.D. (2008). Training neural network classifiers for medical decision making: The effects of imbalanced datasets on classification performance. Neural. Netw..

[25] García-Pedrajas N., Ortiz-Boyer D., García-Pedrajas M.D., Fyfe C. Class imbalance methods for translation initiation site recognition. Proceedings of the International Conference on Industrial, Engineering and Other Applications of Applied Intelligent Systems.

[26] Blagus R., Lusa L. (2010). Class prediction for high-dimensional class-imbalanced data. BMC Bioinform..

[27] Xia S., Xue J.B., Zhang X., Hu H.H., Abe E.M., Rollinson D., Bergquist R., Zhou Y., Li S.Z., Zhou X.N. (2017). Pattern analysis of schistosomiasis prevalence by exploring predictive modeling in Jiangling County, Hubei Province, China. Infect. Dis. Poverty.

[28] Ali Y., Farooq A., Alam T.M., Farooq M.S., Awan M.J., Baig T.I. (2019). Detection of Schistosomiasis Factors Using Association Rule Mining. IEEE Access.

[29] Wrable M., Kulinkina A.V., Liss A., Koch M., Cruz M.S., Biritwum N.-K., Ofosu A., Gute D.M., Kosinski K.C., Naumova E.N. (2019). The use of remotely sensed environmental parameters for spatial and temporal schistosomiasis prediction across climate zones in Ghana. Environ. Monit. Assess..

[30] Gong Y.-F., Zhu L.-Q., Li Y.-L., Zhang L.-J., Xue J.-B., Xia S., Lv S., Xu J., Li S.-Z. (2021). Identification of the high-risk area for schistosomiasis transmission in China based on information value and machine learning: A newly data-driven modeling attempt. Infect. Dis. Poverty.

[31] Van Buuren S. (2018). Flexible Imputation of Missing. https://scholar.google.com/scholar?hl=en&as_sdt=0%2C5&q=Van+Buuren%2C+S.+%282018%29+Flexible+Imputation+of+Missing+Data.+Chapman+and+Hall%2FCRC&btnG=.

[32] Patro S.G.K., Sahu K.K. (2015). Normalisation: A preprocessing stage. arXiv.

[33] Fan Q., Zhu C.J., Xiao J.Y., Wang B.H., Yin L., Xu X.L., Rong F. An application of apriori algorithm in SEER breast cancer data. Proceedings of the 2010 International Conference on Artificial Intelligence and Computational Intelligence.

[34] Shaukat K., Luo S., Abbas N., Mahboob Alam T., Ehtesham Tahir M., Hameed I.A. An analysis of blessed Friday sale at a retail store using classification models. Proceedings of the 4th International Conference on Software Engineering and Information Management (ICSIM 2021).

[35] Joseph J., Badrinath P., Basran G.S., Sahn S.A. (2002). Is albumin gradient or fluid to serum albumin ratio better than the pleural fluid lactate dehydroginase in the diagnostic of separation of pleural effusion?. BMC Pulm. Med..

[36] Alam T.M., Mushtaq M., Shaukat K., Hameed I.A., Sarwar M.U., Luo S. (2021). A Novel Method for Performance Measurement of Public Educational Institutions Using Machine Learning Models. Appl. Sci..

[37] Azhagusundari B., Thanamani A.S. (2013). Feature selection based on information gain. Int. J. Innov. Technol. Explor. Eng..

[38] Hall M. (1999). Correlation-Based Feature Selection for Machine Learning. Doctoral Dissertation.

[39] Wang Y., Makedon F. Application of Relief-F feature filtering algorithm to selecting informative genes for cancer classification using microarray data. Proceedings of the 2004 IEEE Computational Systems Bioinformatics Conference, 2004. CSB 2004.

[40] Mejía-Lavalle M., Sucar E., Arroyo G. Feature selection with a perceptron neural net. Proceedings of the International Workshop on Feature Selection for Data Mining.

[41] Wu X., Kumar V., Quinlan J.R., Ghosh J., Yang Q., Motoda H., McLachlan G.J., Ng A., Liu B., Yu P.S. (2008). Top 10 algorithms in data mining. Knowl. Inf. Syst..

[42] Praveena H.D., Subhas C., Naidu K.R. (2021). Automatic epileptic seizure recognition using reliefF feature selection and long short term memory classifier. J. Ambient Intell. Humaniz. Comput..

[43] Fernández A., Garcia S., Herrera F., Chawla N.V. (2018). SMOTE for learning from imbalanced data: Progress and challenges, marking the 15-year anniversary. J. Artif. Intell. Res..

[44] Mani I., Zhang I. kNN approach to unbalanced data distributions: A case study involving information extraction. Proceedings of the Workshop on Learning from Imbalanced Datasets.

[45] Yen S.J., Lee Y.S. (2009). Cluster-based under-sampling approaches for imbalanced data distributions. Expert Syst. Appl..

[46] Nasir A., Shaukat K., Iqbal Khan K., A. Hameed I., Alam T.M., Luo S. (2021). Trends and Directions of Financial Technology (Fintech) in Society and Environment: A Bibliometric Study. Appl. Sci..

[47] Khushi M., Shaukat K., Alam T.M., Hameed I.A., Uddin S., Luo S., Yang X., Reyes M.C. (2021). A Comparative Performance Analysis of Data Resampling Methods on Imbalance Medical Data. IEEE Access.

[48] Han H., Wang W.Y., Mao B.H. (2005). Borderline-SMOTE: A new over-sampling method in imbalanced data sets learning. Proceedings of the International Conference on Intelligent Computing.

[49] Last F., Douzas G., Bacao F. (2017). Oversampling for Imbalanced Learning Based on K-Means and SMOTE. Inf. Sci..

[50] Douzas G., Bacao F., Sciences F.L.-I. (2018). Improving imbalanced learning through a heuristic oversampling method based on k-means and SMOTE. Inf. Sci..

[51] Friedman J.H. (2001). Greedy function approximation: A gradient boosting machine. Ann. Stat..

[52] Friedman J.H. (2002). Stochastic gradient boosting. Comput. Stat. Data Anal..

[53] Chen T., Guestrin C. XGBoost: A Scalable Tree Boosting System. Proceedings of the 22nd Acm Sigkdd International Conference on Knowledge Discovery and Data Mining.

[54] Ke G., Meng Q., Finley T., Wang T., Chen W., Ma W., Ye Q., Liu T.Y. (2017). LightGBM: A highly efficient gradient boosting decision tree. Adv. Neural Inf. Process. Syst..

[55] Dorogush A.V., Ershov V., Gulin A. (2018). CatBoost: Gradient boosting with categorical features support. arXiv.

[56] Shaukat K., Luo S., Varadharajan V., Hameed I.A., Xu M. (2020). A Survey on Machine Learning Techniques for Cyber Security in the Last Decade. IEEE Access.

[57] Shaukat K., Luo S., Varadharajan V., Hameed I.A., Chen S., Liu D., Li J. (2020). Performance Comparison and Current Challenges of Using Machine Learning Techniques in Cybersecurity. Energies.

[58] Shaukat K., Luo S., Varadharajan V. (2022). A novel method for improving the robustness of deep learning-based malware detectors against adversarial attacks. Eng. Appl. Artif. Intell..

[59] Nasir A., Shaukat K., Khan K.I., Hameed I.A., Alam T.M., Luo S. (2020). What is core and what future holds for blockchain technologies and cryptocurrencies: A bibliometric analysis. IEEE Access.

[60] Ibrar M., Muhammad A.H., Kamran S., Talha M.A., Khaldoon S.K., Ibrahim A.H., Hanan A., Suhuai L. (2022). A Machine Learning-Based Model for Stability Prediction of Decentralized Power Grid Linked with Renewable Energy Resources. Wirel. Commun. Mobile Comput..

[61] Batool D., Shahbaz M., Shahzad Asif H., Shaukat K., Alam T.M., Hameed I.A., Ramzan Z., Waheed A., Aljuaid H., Luo S. (2022). A Hybrid Approach to Tea Crop Yield Prediction Using Simulation Models and Machine Learning. Plants.

[62] Alam T.M., Shaukat K., Hameed I.A., Khan W.A., Sarwar M.U., Iqbal F., Luo S. (2021). A novel framework for prognostic factors identification of malignant mesothelioma through association rule mining. Biomed. Signal Process. Control..

[63] Shaukat K., Luo S., Chen S., Liu D. Cyber threat detection using machine learning techniques: A performance evaluation perspective. Proceedings of the 2020 International Conference on Cyber Warfare and Security (ICCWS).

[64] Shaukat K., Masood N., Khushi M. (2019). A Novel Approach to Data Extraction on Hyperlinked Webpages. Appl. Sci..

[65] Kumar M.R., Vekkot S., Lalitha S., Gupta D., Govindraj V.J., Shaukat K., Alotaibi Y.A., Zakariah M. (2022). Dementia Detection from Speech Using Machine Learning and Deep Learning Architectures. Sensors.

[66] Alam T.M., Shaukat K., Khelifi A., Khan W.A., Raza H.M.E., Idrees M., Luo S., Hameed I.A. (2022). Disease Diagnosis System Using IoT Empowered with Fuzzy Inference System. Comput. Mater. Contin..

[67] Alam T.M., Shaukat K., Mahboob H., Sarwar M.U., Iqbal F., Nasir A., Hameed I.A., Luo S. (2021). A Machine Learning Approach for Identification of Malignant Mesothelioma Etiological Factors in an Imbalanced Dataset. Comput. J..

[68] Shaukat K., Rubab A., Shehzadi I., Iqbal R. (2017). A socio-technological analysis of cyber crime and cyber security in Pakistan. Transylv. Rev..

[69] Shabbir S., Asif M.S., Alam T.M., Ramzan Z. (2021). Early Prediction of Malignant Mesothelioma: An Approach Towards Non-invasive Method. Curr. Bioinform..

[70] Latif M.Z., Shaukat K., Luo S., Hameed I.A., Iqbal F., Alam T.M. Risk Factors Identification of Malignant Mesothelioma: A Data Mining Based Approach. Proceedings of the 2020 International Conference on Electrical, Communication, and Computer Engineering (ICECCE).

